# Uncommon acquired *Gerbode *defect following extensive bicuspid aortic valve endocarditis

**DOI:** 10.1186/1476-7120-10-7

**Published:** 2012-02-23

**Authors:** Hélder Dores, João Abecasis, Regina Ribeiras, José Pedro Neves, Miguel Mendes

**Affiliations:** 1 Laboratório de Ecocardiografia, Serviço de Cardiologia, Hospital de Santa Cruz - Centro Hospitalar de Lisboa Ocidental, Rua Professor Reynaldo dos Santos, 2795-523 Carnaxide, Portugal; 2 Serviço de Cirurgia Cardiotorácica, Hospital de Santa Cruz - Centro Hospitalar de Lisboa Ocidental, Rua Professor Reynaldo dos Santos, 2795-523 Carnaxide, Portugal

**Keywords:** Gerbode defect, Infective endocarditis, Bicuspid aortic valve, Prosthetic leak, Valve replacement

## Abstract

*Gerbode *defect is a rare type of left ventricle to right atrium shunt. It is usually congenital in origin, but acquired cases are also described, mainly following infective endocarditis, valve replacement, trauma or acute myocardial infarction. We report a case of a 50-year-old man who suffered an extensive and complex infective endocarditis involving a bicuspid aortic valve, the mitral-aortic intervalvular fibrosa and the anterior leaflet of the mitral valve. After dual valve replacement and annular reconstruction, a shunt between the left ventricle and the right atrium - *Gerbode *defect, and a severe leak of the mitral prosthesis were detected. Reintervention was performed with successful shunt closure with an autologous pericardial patch and paravalvular leak correction. No major complications occurred denying the immediate post-surgery period and the follow-up at the first year was uneventful.

## Background

The uncommon intercavitary shunt between the left ventricle (LV) to right atrium (RA), known as *Gerbode *defect (GD) has been mainly described as congenital in origin, representing less than 1% of all congenital heart defects. However there are also few cases reported as acquired, namely after complicated infective endocarditis (IE) [1]. Its clinical spectrum depends on the underlying etiology and the size of the defect. In small ones, it may be well tolerated without symptoms or clinical signs, except for an associated murmur. Larger communications lead to volume overload, chamber enlargement and eventually heart failure.

Despite being a rare intercavitary shunt, its nature, with an abnormal flow towards an anterior cardiac chamber, shall be revealed by a comprehensive detailed transthoracic echocardiography (TTE), obviating the need for other imaging modalities.

We report an unusual acquired GD developing in the clinical workup of a complicated case of aortic valve IE. TTE was sufficient both to establish the diagnosis and to decide its management.

## Case presentation

We describe a clinical case of a 50-year-old man admitted to the emergency department due to recurrent fever, tiredness and poliarthralgia with one month of evolution. He was previously followed at our institution for a bicuspid aortic valve with moderate aortic regurgitation, mild LV dilatation and preserved LV ejection fraction. Physical examination was positive for fever (38.4°C), high pulse pressure, a 3/6 holosystolic murmur over the precordium and bilateral pulmonary rales. Laboratorial profile showed a normocytic normochromic anaemia (Hb: 10.8 g/L) and increased inflammatory markers (C-reactive protein: 15.2 mg/dl, sedimentation velocity: 69 mm/h, leucocytes: 11500/L). The 12-lead electrocardiogram revealed a first-degree atrioventricular block, prolonged at 260 ms, not previously known.

**Figure 1:**
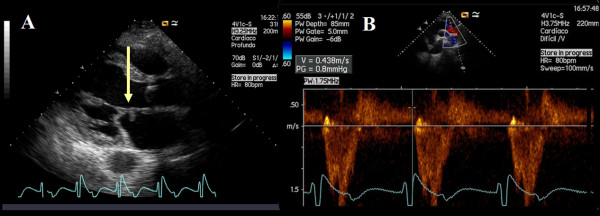
**Transthoracic echocardiogram - parasternal long-axis view with evidence of a nodular image attached to the atrial side of the anterior leaflet of the mitral valve (A, arrow) and a holodiastolic flow reversal in the descending thoracic aorta recorded from a suprasternal notch window with terminal velocity > 20 cm/s (B) supporting severe aortic regurgitation**.

For the clinical suspicion of an infective IE a TTE was performed, revealing an echogenic, pediculated and mobile mass attached to the atrial surface of the anterior mitral valve leaflet. Both severe aortic and mitral regurgitation were present (Figure 1 and Additional files: clip S1 and Clip S2). In fact, two mitral valve regurgitation jets were visualized, one of them leading to the suspicion of leaflet perforation (Additional file 3: Clip S3).

**Figure 2:**
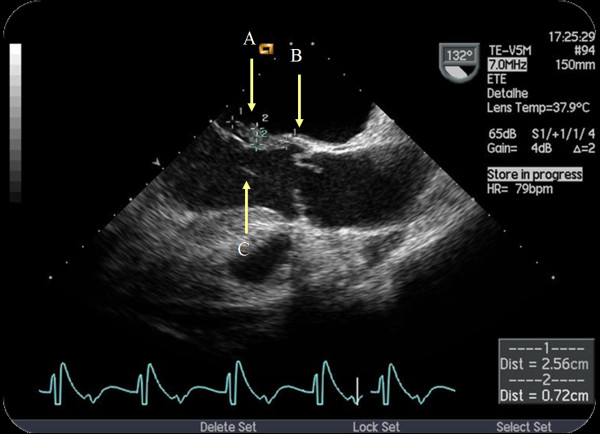
**Figure 2 and Clip S4 Transoesophageal long-axis view of the aortic valve**. Visualization of a long vegetation (**A**) (larger axis: 26 mm) on the atrial side of the mitral valve, with insertion on the aortic ring (**B**) and reduced. It was still possible to visualize the morphological distortion of the aortic cusps, its eccentric closure (bicuspid) as well as a filamentar image in the left ventricular outflow tract (LVOT) (**C**), probably in concordance with other vegetation (**C**). There was a large aortic regurgitation jet, anteriorly directed to the anterior leaflet of the mitral valve. In relation with mitral valve involvement by the vegetation, but equally as possible injury of the aortic jet, there was a perforation of the anterior leaflet with an important regurgitation jet. ECG recording was notable for a large atrio-ventricular conduction delay.

In order to confirm the diagnosis, assess aortic valve involvement and exclude local complications, a transoesophageal echocardiogram (TEE) was done. It confirmed anterior mitral leaflet perforation, also revealing a long vegetation involving the origin of the anterior cusp of the aortic valve, the aortic ring, the mitro-aortic intervalvular fibrosa and the anterior leaflet of the mitral valve (Figure 2 and Additional file 4: Clip S4).

Blood cultures were positive for *Streptoccocus anginosous *and previous empirical antibiotherapy was changed accordingly. Although clinical evolution was favorable, at the nineth day of admission the patient was submitted to mitral and aortic valve replacement due to: heart failure at admission with severe mitral and aortic regurgitation; > 15 mm vegetation length; local complications with mitro-aortic fibrosa involvement and anterior mitral leaflet perforation. Two mechanical valves, St Jude 29 and St Jude 23, were implanted at mitral and aortic position, respectively. Both aortic valve annulus and membranous septum reconstruction with an autologous pericardium patch were performed - *Manouguian *surgery.

**Figure 3:**
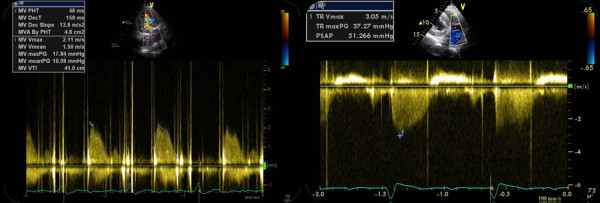
**Postoperative transthoracic ecocardiogram showing an abnormally high gradient of the mitral prosthesis (maximal 18 mmHg and mean 10 mmHg), with a deceleration time (< 130 ms), possibly correlated with an increased flow across the prothesis (left panel)**. High pulmonary artery systolic pressure was assessed (51 mmHg) (right panel).

Immediate postoperative period was uneventful albeit new pathological findings at the TTE performed ten days after surgery. This exam revealed an abnormal systolic jet between the LV and the RA at parasternal short axis view, even without changing colour baseline or scale, above the tricuspid valve, without hemodynamic compromise (Additional files: Clip S5 and S6). There was also an abnormally high gradient of the mitral prosthesis and high pulmonary artery systolic pressure (Figure 3). The TEE study made afterwards confirmed the presence of an abnormal systolic jet entering the RA and revealed an anterior moderate-to-severe peri-prosthetic mitral leak (Additional files: Clip S7 and S8). Surgical correction was achieved with closure of the intercavitary defect with an autologous pericardial patch and prosthetic leak suture.

The patient was dismissed without major complications and with normal prosthesis evaluation. At the first year after discharge, clinical follow-up was uneventful and echocardiographic assessment did not show any additional abnormal finding (Additional file 9: Clip S9).

## Discussion

GD was described for the first time as a very rare congenital defect in 1958. Acquired cases were subsequently reported, mainly in association with IE but also after valvular surgery, thoracic trauma, repaired atrioventricular septal defect and mechanical complications of ischemic heart disease [2-5].

The mechanism explaining acquired GD is not clearly known. Following IE, bacterial infection of the subannular region with involvement of the high membranous interventricular septum has been suggested as a possible cause for the acquired shunt [6,7]. *Staphylococcus aureus *infection is more frequently involved, possibly due to its pathogenic aggressiveness and capability to cause local complications.

Early after valve replacement GD can be related both with the extension of the valvular debridement and the erosion of the membranous septum by the rigid prosthetic ring.

GD is anatomically possible because the normal tricuspid valve is more apically displaced than the mitral valve. Two presentations of GD were described by *Riemenschneider *and *Moss *in 1967, according to the insertion of the septal leaflet of the tricuspid valve, which divides the membranous septum into two portions - interventricular, below the valve, and atrioventricular, above the valve. This anatomical substract differentiates congenital from acquired forms of GD. In the first case, the shunt is usually found below the insertion of the tricuspid valve, with a communication between LV and RA through the septal leaflet. These congenital shunts often coexist with tricuspid valve abnormalities. In the acquired form, the communication is above the insertion point of the tricuspid valve leaflet [8,9].

Preoperative diagnosis is often difficult. One of the hallmarks of GD is the high Doppler gradient between LV and RA on echocardiogram. However, it can be mistakenly interpreted as a tricuspid regurgitation jet and in this case color Doppler can be useful for delineating the origin and direction of the different jets [9]. Furthermore modified parasternal short-axis and sub-costal views allowing simultaneous RA and LV outflow tract visualization have to be tried when a GD is suspected, eventually with color settings adjustments for the detection of lower velocity shunts. Despite having lower spatial resolution than TEE, TTE may be more sensitive for its diagnosis as these jets are anteriorly directed, towards the RA [10]. In the setting of an extensive and destructive IE with an agressive and modified surgical approach involving patches and enlarged tissue debridement, a comprehensive follow-up TTE has to be performed. Particular detailed evaluation upon atrioventricular level, LV outflow tract and intervalvular fibrosa should be attempted.

In small defects, direct closure is possible, but larger ones require a patch of pericardium. As an alternative to surgery, there are reported cases of percutaneous closure with septal occluders, while not in the context of prosthetic valve implantation [11]. Atrioventricular block as a possible complication of the correction should be taken into account as it occurs in approximately 4% of the patients [8].

## Conclusions

In our case, the suspicion of GD was raised through clinical and echocardiographic data correlation. Extensive complex surgical intervention involving not only aortic annulus but also the fibrous skeleton of the heart in the setting of a complicated IE should raise the suspicion and may easily explain unusual anatomic findings. Moreover the specific lesion diagnosis could be made by TTE, even considering that in this patient TEE was also very important for the confirmation of a more common complication after IE surgery: prosthetic leakage.

We found this case peculiar for being a rare situation of an acquired GD in a patient with bicuspid aortic valve IE. To our knowledge this is the second reported case [5], in which a focused and detailed TTE played a central role in both diagnosis and management.

## Competing interests

The authors declare that they have no competing interests (there are not disclosures of any relationship with industry).

## Authors' contributions

All other authors were directly involved in patient management and written report. HD is a cardiology resident at the Cardiology Department of Hospital de Santa Cruz, Carnaxide - Portugal (the corresponding author of the manuscript), being particularly involved in the clinical assessment and follow-up of the patient presented in the reported case. He is also responsible for the main written case report. JA and RR were responsible for the first echocardiographic evaluation and diagnosis. JPN was the cardiothoracic surgeon being the director in chief of the Cardiothoracic Surgery Department. MM is the head director of the Cardiology Department. All authors read and approved the manuscript. The present report has not been previously published.

## Supplementary Material

Additional file 1 **Clip S1**. Parasternal long axis view revealing an echogenic, pediculated and mobile mass attached to the atrial side of the anterior leaflet of the mitral valve. Click here for file

Additional file 2 **Clip S2**. Color Doppler imaging by suprasternal view depicting diastolic flow reversal at the descending thoracic aorta. Click here for file

Additional file 3 **Clip S3**. Transthoracic apical view showing two mitral valve regurgitation jets, one of them in possible correlation with an anterior leaflet perforation. Click here for file

Additional file 4 **Figure **2**and Clip S4**. Transoesophageal long-axis view of the aortic valve. Visualization of a long vegetation (**A) **(larger axis: 26 mm) on the atrial side of the mitral valve, with insertion on the aortic ring (**B**) and reduced. It was still possible to visualize the morphological distortion of the aortic cusps, its eccentric closure (bicuspid) as well as a filamentar image in the left ventricular outflow tract (LVOT) (**C**), probably in concordance with other vegetation (**C**). There was a large aortic regurgitation jet, anteriorly directed to the anterior leaflet of the mitral valve. In relation with mitral valve involvement by the vegetation, but equally as possible injury of the aortic jet, there was a perforation of the anterior leaflet with an important regurgitation jet. ECG recording was notable for a large atrio-ventricular conduction delay. Click here for file

Additional file 5 **Clip S5 and Clip S6**. Abnormal systolic jet between the left ventricle and the right atrium above the tricuspid valve (parasternal short axis view - Clip S5 and subcostal four chambers view - Clip S6). Click here for file

Additional file 6 **Clip S5 and Clip S6**. Abnormal systolic jet between the left ventricle and the right atrium above the tricuspid valve (parasternal short axis view - Clip S5 and subcostal four chambers view - Clip S6). Click here for file

Additional file 7 **Clip S7 and Clip S8**. An abnormal systolic jet entering the right atrium was seen at a high esophageal view (Clip S7). A moderate-to-severe peri-prosthetic leak was also evident (Clip S8). Click here for file

Additional file 8 **Clip S7 and Clip S8**. An abnormal systolic jet entering the right atrium was seen at a high esophageal view (Clip S7). A moderate-to-severe peri-prosthetic leak was also evident (Clip S8). Click here for file

Additional file 9 **Clip S9**. Transthoracic echocardiogram - parasternal short axis view performed during the follow-up showing the absence of the shunt between the left ventricle and the right atrium. Click here for file
